# Direct observation of muonic molecules in resonance states critical to muon catalyzed fusion

**DOI:** 10.1126/sciadv.aed3321

**Published:** 2026-04-15

**Authors:** Y. Toyama, T. Azuma, D. A. Bennett, W. B. Doriese, M. S. Durkin, J. W. Fowler, J. D. Gard, T. Hashimoto, R. Hayakawa, Y. Ichinohe, K. Ishida, S. Kanda, N. Kawamura, Y. Kino, R. Konishi, Y. Miyake, K. M. Morgan, R. Nakashima, H. Natori, H. Noda, G.C. O’Neil, S. Okada, T. Okumura, K. Okutsu, C. D. Reintsema, K. Sasaki, T. Sato, D. R. Schmidt, K. Shimomura, P. Strasser, D. S. Swetz, T. Takahashi, M. Tampo, H. Tatsuno, J. N. Ullom, I. Umegaki, S. Watanabe, S. Yamada, T. Yamashita

**Affiliations:** ^1^ Center for Muon Science and Technology, Chubu University, Kasugai 487-8501, Japan.; ^2^ Atomic, Molecular and Optical Physics Laboratory, RIKEN, Wako 351-0198, Japan.; ^3^ WPI-QUP, International Center for Quantum-field Measurement Systems for Studies of the Universe and Particles, High Energy Accelerator Research Organization (KEK), Tsukuba 305-0801, Japan.; ^4^ National Institute of Standards and Technology, Boulder, CO 80305, USA.; ^5^ University of Colorado, Boulder, CO 80309, USA.; ^6^ RIKEN Pioneering Research Institute, RIKEN, Wako 351-0198, Japan.; ^7^ Nishina Center for Accelerator-Based Science, RIKEN, Wako 351-0198, Japan.; ^8^ High Energy Accelerator Research Organization (KEK), Tsukuba 305-0801, Japan.; ^9^ Department of Chemistry, Tohoku University, Sendai 980-8578, Japan.; ^10^ Astronomical Institute, Tohoku University, Sendai 980-8578, Japan.; ^11^ Department of Mathematical and Physical Sciences, Chubu University, Kasugai 487-8501, Japan.; ^12^ National Institute for Fusion Science (NIFS), Toki 509-5292, Japan.; ^13^ Department of Chemistry, Tokyo Metropolitan University, Hachioji 192-0397, Japan.; ^14^ Department of Physics, Meiji University, Kawaski 214-8571, Japan.; ^15^ Kavli IPMU (WPI), The University of Tokyo, Kashiwa 277-8583, Japan.; ^16^ Department of Physics, Tokyo Metropolitan University, Hachioji 192-0397, Japan.; ^17^ Department of Space Astronomy and Astrophysics, Institute of Space and Astronautical Science (ISAS), Sagamihara 252-5210, Japan.; ^18^ Department of Physics, Rikkyo University, Tokyo 171-8501, Japan.

## Abstract

Muon catalyzed fusion (μCF) is a plasma-free process in which the formation of muonic hydrogen molecules precedes and enables fusion between their constituent nuclei. Despite decades of study, the reaction dynamics of μCF remain elusive. Recent theories predict that resonance states of the muonic molecules play a key role and that these states can be probed using x-ray techniques. Using an array of transition-edge sensor microcalorimeters with 10-fold improved energy resolution compared to conventional silicon detectors, we observed x-rays from resonance states of muonic deuterium molecules despite an intense background. The spectrum is well explained by high-precision calculations incorporating the vibrational states. This work identifies the long-overlooked resonance state pathway as crucial in μCF and provides the direct evidence of the efficient muonic molecular formation.

## Teaser

High-resolution x-ray spectroscopy with a cryogenic detector reveals long-ignored resonance states driving muon catalyzed fusion.

## INTRODUCTION

Muon catalyzed fusion (μCF) is a type of nuclear fusion in which a muon drives a distinctive cyclic reaction that intricately couples atomic, molecular, and nuclear processes. A negatively charged muon, whose mass is 207 times that of an electron, can bind two hydrogen isotope nuclei into a compact muonic molecular state. In this state, the internuclear separation is reduced to nearly 1/200 of that in an ordinary hydrogen molecule, enabling intramolecular fusion to occur efficiently. After fusion, the muon is released and can participate in the formation of another muonic molecule. This series of reactions is repeated multiple times within the muon’s 2.2 μs lifetime, with the muon thus acting as a catalyst.

μCF was first proposed theoretically in the late 1940s (*1*, *2*) and extensively investigated from the 1980s to around 2000 (*3*–*9*), aiming at an application to energy production. In recent years, it has attracted renewed attention in light of new potential applications. The monochromatic neutrons emitted following nuclear fusion can be used to transmute long-lived fission products in high-level nuclear waste (*10*), the treatment of which remains a major societal challenge. The muons released upon the nuclear fusion can also be used as a cold muon source (*11*) that finds applications to material science and fundamental physics (*12*, *13*).

For the purpose of energy production by μCF, ~300 fusion reactions per muon are required to reach scientific breakeven. Among the possible isotope combinations, deuterium-tritium (D-T) mixtures are the most favorable, and multiple experiments have achieved more than 100 reactions (*14*). The overall efficiency of μCF is mainly limited by two factors: muon loss due to αμ sticking, where the muon adheres to the alpha particle generated as a fusion product, and the formation rate of muonic molecules (*3*). While experiments with solid D-T targets have provided hints for mitigating muon loss (*15*), a complete theoretical understanding of solid-state effects is still lacking (*16*).

**Fig. 1.**
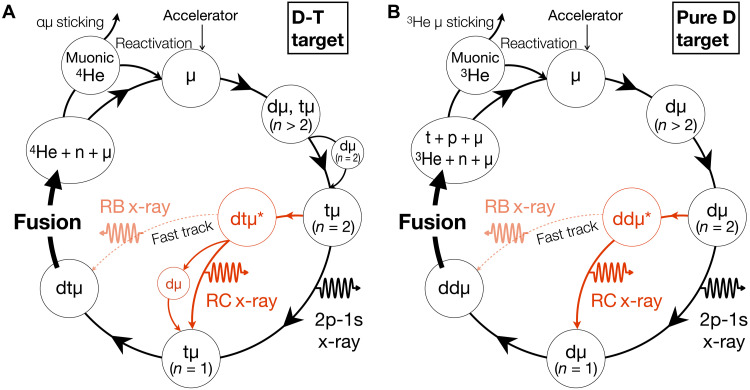
μCF cycle and x-ray emission from dissociation of muonic molecules in resonance states. (**A**) Schematic representation of the μCF cycle in a D-T mixture target. A muon provided by an accelerator initially forms a highly excited muonic atom (*n* ∼ 14). The muonic atom cascades to lower states (*n* = 1, 2) and forms muonic molecules. The *n* = 2 state is not necessarily traversed; however, under conditions of solid density, most of the transitions are expected to proceed through it. X-rays from dissociating resonance states, referred to as RC transitions, are emitted when a muonic molecular resonance state breaks up into its constituent muonic atom and nucleus. RB x-ray denotes a RB transition, where the muonic molecular resonance state decays into a bound ground state, emitting monochromatic x-rays. (**B**) μCF cycles in a pure deuterium target. Although the fusion rate in D_2_ target is slow, the process can be understood as a mechanism similar to that in D-T mixture target.

Figure 1 illustrates the μCF cycles in two representative cases: a D-T mixture target and a pure deuterium target. In the D-T cycle (Fig. 1A), the muon provided by an accelerator initially forms an excited muonic tritium atom (tμ). The tμ cascades to the 1s state (*17*), eventually forming a muonic molecule (dtμ) through collisions with D_2_ molecules (*18*). Once the dtμ molecule is formed, intramolecular fusion occurs almost instantaneously [at a reaction rate of 10^12^ s^−1^; see (*6*) and references therein], which releases the muon for further catalytic cycles. In addition to dtμ fusion, muonic deuterium molecules (ddμ) can also be formed, leading to fusion in a pure deuterium target, as illustrated in Fig. 1B.

One of the long-standing questions in μCF kinetics relates to the spontaneous formation of muonic molecules in resonance states, dtμ* (*19*, *20*), which has been ignored in the analysis of the μCF experiments. As highlighted by the red paths in Fig. 1A, the dtμ* state may form from tμ(*n* = 2) and dissociates into its constituent muonic atom and nucleus, i.e., the continuum states.

A recent theoretical study that takes into account all isotopologs of muonic molecules in resonance states has brought conventional μCF kinetic models into good agreement with experimentally observed cycle rates at various temperatures (*21*). To date, however, no direct experimental evidence has confirmed the existence of these resonance states. In earlier experiments using a hydrogen (H_2_) target, pμ atoms with kinetic energies of 0.9 keV, which could be originated from ppμ*, were observed (*22*), although theoretical studies have supported other mechanisms (*23*). While observations of a delayed x-ray component suggested the existence of resonant states of muonic deuterium molecules (*24*), the direct spectral separation of the molecular x-rays from atomic lines has not yet been achieved.

A more direct evidence of the muonic molecules formed in resonance states will be obtained by measuring the x-rays emitted upon the dissociation, i.e., the resonance-to-continuum (RC) transition. According to precise few-body calculations, both dtμ* and ddμ* are predicted to emit RC x-rays with state-dependent spectra upon dissociation (*25*, *26*). More recently, Yamashita *et al.* (*27*) has provided detailed predictions of RC x-ray spectra and, in addition, introduced the possibility of resonance-to-bound (RB) x-rays that are emitted when certain resonance states decay directly into bound states, as indicated in Fig. 1. These x-rays provide direct insights into the quantum structure of the resonance states, and their energies are expected to fall in the 1.6- to 2.0-keV range for ddμ*; however, the limited resolution of conventional x-ray detectors has so far prevented their separation from the intense 2p-1s transition at 2.0 keV of dμ atoms (*28*–*30*).

In this study, we experimentally verified the existence of muonic molecules in resonance states (ddμ*) by using a solid deuterium target and a state-of-the-art, high-resolution x-ray detector. The ddμ* system was selected due to its spectral simplicity as a homonuclear molecule, in contrast to the more complex dtμ* case, where overlapping signals from dμ, tμ, ddμ*, and ttμ* obscure clear identification. Our results demonstrate that precise x-ray spectroscopy enables quantum state–resolved analysis of μCF cycles.

## RESULTS

### Experimental overview

The experiment was conducted at the D2 muon beamline of the Materials and Life Science Experimental Facility (MLF) at Japan Proton Accelerator Research Complex (J-PARC) (*31*) using a pulsed negative muon beam. Muons were stopped in a cryogenically solidified deuterium target (*32*, *33*), where the high density enhances the stopping efficiency and, in addition, increases the population of dμ(*n* = 2) atoms (*23*), thereby improving the probability of forming ddμ*.

To detect x-rays emitted upon their dissociation with high precision, we used an array of superconducting transition-edge sensor (TES) microcalorimeters developed by the National Institute of Standards and Technology (*34*, *35*). The TES detector enabled energy resolution better than 10 eV in the 1.6- to 2.0-keV range, where the dissociation signals are expected. A detailed description of the experimental setup is provided in Materials and Methods. The energy calibration of the TES detector and the data analysis are described in the Supplementary Materials.

### Obtained x-ray spectrum

**Fig. 2.**
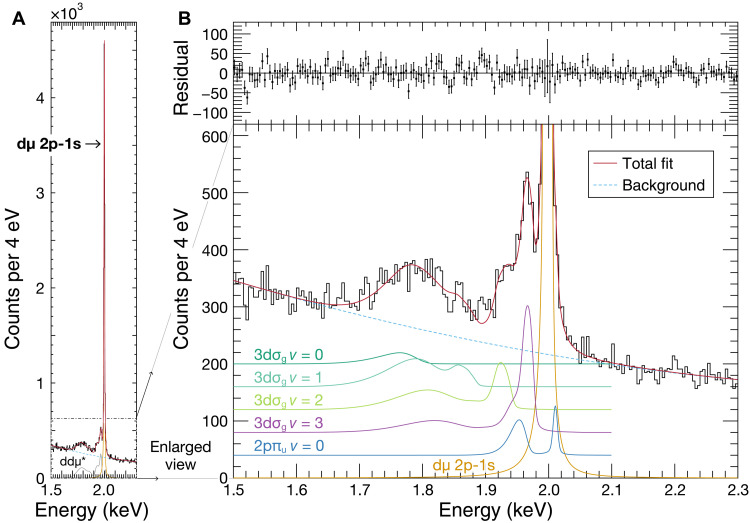
X-ray spectrum associated with the dissociation of resonance states in the muonic deuterium molecule. (**A**) Full x-ray spectrum measured using a solid D_2_ target. The sharp peak near 2.0 keV corresponds to the monochromatic dμ 2p-1s transition. The total fit to the data is shown as a solid red line, consisting of the dμ 2p-1s peak (orange line), bremsstrahlung background (dashed blue line), and the summed contribution from the dissociation of ddμ* resonance states (gray line). (**B**) Enlarged view of the spectral region enclosed by the dot-dashed rectangle in (A), showing the same fit components, except that the ddμ* contribution is decomposed into individual vibrational components, each shown as a colored solid line with offsets for better visibility. The TES response function was modeled using a combination of a Gaussian function for its main peak and an asymmetric Lorentzian function for its tail structure to properly represent its whole structure. The residuals between the data and the fit are shown in the top subpanel. The reduced chi-square is χ^2^/ndf = 209.6/193 = 1.086, where ndf denotes number of degrees of freedom.

Figure 2 presents the x-ray spectrum from the solid D_2_ target at the timing of the muon beam irradiation. The sharp peak at 2.00 keV corresponds to the 2p-1s transition of dμ atoms, appearing against a continuous background from bremsstrahlung radiation produced by electrons emitted during muon decay. A distinct structure was observed on the low-energy side of the intense dμ 2p-1s peak, reflecting the wave function of dissociating ddμ* states. The spectrum was fitted using three components: dμ 2p-1s transition peak, a continuous bremsstrahlung background, and the x-ray spectra resulting from dissociation of ddμ*, as shown in Fig. 2A.

To account for the distortion caused by energetic charged particles produced by the muon beam (*36*), the in-beam response function of the TES was derived from the dμ 2p-1s peak, achieving an energy resolution of 8.3 ± 0.1 eV (full width at half maximum) at 2 keV. Because the dμ 2p-1s peak partially overlaps with the ddμ* dissociation spectrum, the response function and the spectrum itself had to be determined self-consistently. This was achieved through iterative fitting, yielding converged results for both components. A more detailed description of the fitting is provided in the Supplementary Materials.

### Quantum structure of ddμ* and associated x-ray spectra

**Fig. 3.**
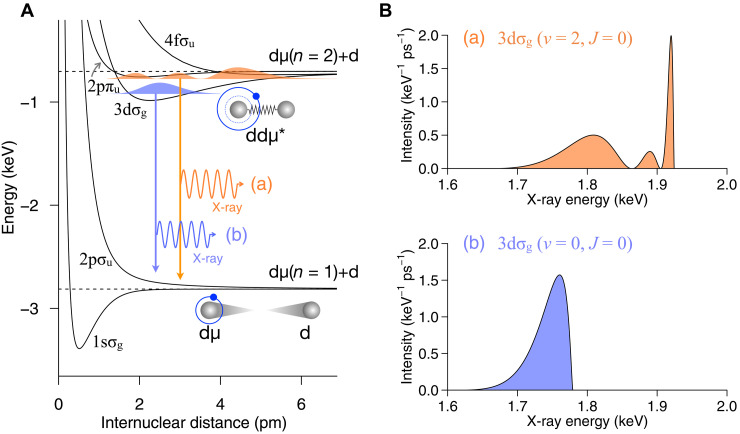
Energy potential diagram and associated x-ray spectra of ddμ molecule. (**A**) Conceptual energy potential diagram for ddμ molecule, highlighting the resonance state ddμ* and their associated RC x-rays under the adiabatic approximation. The horizontal axis represents the internuclear distance between the deuterons in ddμ molecule. The dashed black lines indicate the energy levels corresponding to dμ(*n* = 2) + d (top) and dμ(*n* = 1) + d (bottom). The colored filled areas represent the probability density reflecting the wavefunction of the resonance states. (**B**) Examples of calculated RC x-ray spectra (*27*) with (a) and (b) corresponding to the different resonance states depicted in (A). Each resonance emits x-rays that show a distribution with characteristic nodes depending on the wavefunction structure.

Figure 3A represents a conceptual energy potential diagram for the ddμ molecule as a function of the internuclear distance. In comparison with ordinary $D_{2}^{+}$, the ~200-fold heavier muon compresses the internuclear distance by ~1/200 and raises the energy scale by two orders of magnitude. This leads to a large zero-point energy in ddμ*, resulting in widely spaced vibrational levels. The ddμ* are quasi-bound states in the 3dσ_g_, 4fσ_u_, or 2pπ_u_ adiabatic potential energy curves. When ddμ* spontaneously dissociates into the 2pσ_u_ state, an x-ray is emitted upon dissociation according to the reaction

$$\text{dd}\mu^{*}\to \mathrm{d\mu}\left(1s\right)+d+\gamma$$ (1)

where γ stands for the x-ray. Because the muon mass is comparable to the nuclear mass, the Born-Oppenheimer approximation is inadequate to quantitatively predict the x-ray spectra. Therefore, we carried out precise few-body calculations (*27*) to quantify the x-ray spectra from each ddμ* state.

Figure 3B shows examples of RC x-ray spectra originating from the 3dσ_g_ resonance state with rotational quantum number *J* = 0 and vibrational quantum numbers *v* = 2, 0. The emitted x-ray energy varies depending on the separation between the two nuclei, known as a Frank-Condon transition. Vibrational excitations introduce node structures in the radial distribution function of two nuclei, which are reflected in the x-ray spectra. In addition, a recent precise few-body calculation (*27*) has suggested an alternative pathway in dtμ* and ddμ*, where certain resonance states with radial distributions similar to a bound state can decay directly into bound states via RB transitions, as indicated by the RB x-ray in Fig. 1A. Possible signatures of these RB transitions are also examined in our spectral analysis.

### Fitting of x-ray spectrum

The ddμ* x-ray spectrum was represented by a sum of the theoretically calculated spectra corresponding to individual quantum states. Each state was characterized by *J* and *v* in 2pπ_u_, 4fσ_u_, and 3dσ_g_ series (*27*). The contribution of each state was estimated by chi-square fitting to the measured spectrum, taking into account the TES detector response and the x-ray window transmittance. Figure 2B shows an enlarged view of the x-ray spectrum from the ddμ* dissociation, with the total fit function as a red solid line. The continuous background was assumed to follow a quadratic function, indicated by the blue dashed line. The residual between the histogram and the fit function is displayed in the top panel.

Each component of the total ddμ* spectrum is shown in a different color in Fig. 2B. The *J* distribution of 3dσ_g_ series was assumed to follow a statistical weight of *W*(*J*) = 2*J* + 1, where *J* ∈ {0, 1, 2, 3}. The data are well reproduced by a ddμ* spectrum with lower vibrational states: 3dσ_g_ at *v* ≤ 3 and 2pπ_u_ at *v* = 0, *J* = 1. The x-rays from the resonance states of 4fσ_u_ series that range from 1.96 to 1.99 keV were not resolved from the dμ 2p-1s peak. The 1.96-keV peak corresponds to the *v* = 3 state of the 3dσ_g_ series, while the broad feature observed around 1.8 keV is primarily attributed to the *v* = 2 and *v* = 1 states of the 3dσ_g_ series. The 2pπ_u_ state with *v* = 0, *J* = 1 is theoretically predicted to have a 30% probability of decaying to the ground state of ddμ via the emission of a single 2.01-keV photon corresponding to the RB x-ray (*27*). This RB x-ray was incorporated into the spectral fit by fixing the ratio of the RC x-ray and RB x-ray.

Table 1 presents the fraction of each state relative to the total x-ray signal from dμ 2p-1s and ddμ* transitions, with corrections of the energy dependence of x-ray transmittance. The ratio of 2pπ_u_ at *v* = 0 includes the RB x-ray contribution as mentioned above. In this analysis, all relevant vibrational resonance states populated by Auger transition following the Vesman mechanism are fully accounted for as discussed in the following section.

**Table 1.** Fraction of each state to the total x-ray signal from dμ and ddμ*. *v* represents vibrational quantum number. The sum of fractions was normalized to 100. Sources of systematic uncertainties are discussed in the Supplementary Materials.

Parent state of x-ray	*v*	Fraction	Uncertainties	
			(stat)	(syst)
dμ (2p)	–	61.0	0.6	2.5
ddμ*(3dσ_g_)	0	2.6	1.0	0.8
	1	10.2	1.4	2.8
	2	10.2	1.0	0.9
	3	12.1	0.7	2.3
ddμ*(2pπ_u_)	0	4.0	0.6	1.3

## DISCUSSION

The x-ray spectrum in Fig. 2 is in good agreement with the sum of the calculated x-ray spectra emitted upon dissociation of ddμ* in lower vibrational states, supporting a so-called Vesman mechanism for resonant molecular formation. The Vesman mechanism was originally proposed for ddμ in the bound state as

$$\mathrm{dd\mu}\left(n=1\right)+D_{2}\to \left[\left(\mathrm{dd\mu}\right)e-\text{de}\right]$$ (2)

where e denotes an electron. The [(ddμ)e − de] represents a D_2_-like molecule in which one of the nuclei is replaced by a quasi-nucleus ddμ, carrying +1 charge. This reaction is substantially enhanced when the sum of the collision energy and the binding energy of ddμ in its excited state matches the excitation energy of the host molecule [(ddμ)e − de]. This energy-matching condition is commonly referred to as the Feshbach resonance. The Vesman mechanism is also effective in the formation of dtμ molecules, which was measured by the time-of-flight of tμ atoms with a kinetic energy of ∼0.4 eV (*37*).

The ddμ* may form through a reaction mechanism analogous to that of the standard ddμ formation

$$\mathrm{d\mu}\left(n=2\right)+D_{2}\to \left[{\left(\mathrm{dd\mu}\right)}^{*}e-\text{de}\right]$$ (3)

The excess energy of ddμ* formation is transferred to the internuclear motion of the host molecule. This energy transfer initially populates the ddμ* in high vibrational resonance states (*v* ≥ 7), which subsequently cascade to lower vibrational levels via the Auger transition (*19*, *38*). The Auger transition rates for these resonance states exceed radiative dissociation rates by several orders of magnitude. Because the ionization energy of a D_2_ molecule is 15.4 eV, only muonic molecular states with binding energies larger than this threshold energy are formed by the Auger transition. These supposed processes constrain the ddμ* states that emit x-rays to be *v* = 0, 1, 2, and 3 for the 3dσ_g_ series, *v* = 0, *J* = 1 for the 2pπ_u_ series, and *v* = 0 for the 4fσ_u_ series, all of which have binding energy larger than 15.4 eV. As shown in the previous section, the observed x-ray spectrum was consistent with these ddμ* states.

Detecting x-rays emitted upon dissociation of ddμ* represents a milestone in the study of μCF. Until now, μCF experiments have primarily relied on detecting fusion products such as neutrons, protons, helium nuclei, and x-rays emitted from muonic helium formed via αμ sticking. While these signals provide valuable information, they only offer indirect clues to the reaction mechanism. Our results present the first direct observation of molecular dynamics in μCF, shedding light on the quantum states governing the reaction, particularly the population distribution of muonic atoms in excited states.

An outcome of our experiment is the precisely measured intensity ratio of x-rays emitted upon dissociation of ddμ* to the dμ 2p-1s transition, determined to be 0.64 ± 0.03 (stat) ± 0.05 (syst). The ddμ* formation competes with x-ray emission of dμ(2p) and rapid Stark mixing between dμ(2s) and dμ(2p). Thus, the x-ray intensity ratio suggests that the ddμ* formation rate is comparable to the dμ(2p) de-excitation rate, 1.2 × 10^11^ s^−1^. Such a high formation rate can be supported only by the Vesman mechanism. Furthermore, this intensity ratio provides a constraint on kinetics models of muonic atoms, such as those described in (*39*). In particular, the dμ(2s) kinetic energy distribution, which has been discussed in several theoretical studies (*40*–*42*), is sensitive to this intensity ratio.

Taking into account the contribution of dissociation without emitting an x-ray, ddμ* → dμ(1s) + d, we conclude that nearly half of the muons in the μCF cycle take a previously unexplored path via the ddμ* resonance, necessitating a reexamination of the conventional μCF analysis. For example, these resonance states must be considered when interpreting the most recent temperature-dependent study on μCF, which reported promising signs of enhanced μCF performance in a solid D-T mixture (*15*).

The observed x-ray spectrum does not exclude the existence of the RB x-rays. This direct transition, referred to as the fast track pathway, bypasses the conventional steps of muon transfer and muonic molecule formation. Whereas typical dtμ formation occurs at a rate of 10^8^ s^−1^ at liquid hydrogen density, the RB process is predicted to proceed at rates exceeding 10^10^ s^−1^, offering a substantially faster route that could greatly enhance the overall μCF cycle rate. Our TES detector may enable the direct identification of monochromatic x-rays from this mechanism in the dtμ* system, providing compelling evidence for its presence and guiding the search for target conditions that enhance its formation.

Our TES detector can be also adopted to investigate αμ sticking process in future experiments. Observing the Doppler broadening of the 8.2-keV x-rays from muonic helium formed after αμ sticking (initial kinetic energy of 3.5 MeV) (*43*, *44*) offers insights into the αμ slowing-down process and allows investigation of the reactivation mechanism. Although the broadening was not measured in this experiment due to limited beam time, the TES detector is capable of measuring it and serves as a powerful tool for exploring the target conditions to minimize muon loss during the μCF cycles.

This study presents the first direct observation of quantum state–resolved dynamics of muonic molecules in their resonance states. Muonic molecules have long been inferred indirectly from fusion products. Unlike bound-state muonic molecules, which fuse promptly without x-ray emission, these resonance states emit x-rays during dissociation, serving as clear fingerprints of their internal quantum structure and providing direct confirmation of muonic molecule formation.

It marks a major advance in our understanding of μCF. The process via the resonance states, long neglected in the analysis of the μCF experimental data, is found to be equally relevant to the conventional process. In addition, the reconstructed x-ray spectra and x-ray ratio of ddμ* to dμ elucidate the efficient muonic molecular formation process via the Vesman mechanism, followed by Auger transitions, which has been previously obscured.

This breakthrough was enabled by TES detectors, which provide over 10-fold better energy resolution than conventional devices and now operate reliably in accelerator environments. Looking ahead to experiments with D-T targets, this technology offers a practical means to tackle major challenges in μCF, including increasing catalytic cycles and reducing αμ sticking.

## MATERIALS AND METHODS

**Fig. 4.**
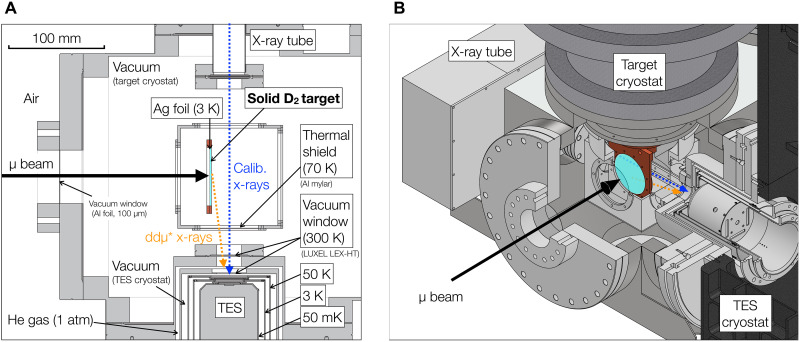
Experimental setup. (**A**) Cross-sectional view of the target chamber and TES detector. (**B**) Schematic of the solid D_2_ target and TES detector. The muon beam is stopped at the D_2_ target on a silver (Ag) foil at 3 K. X-rays from both the target and the x-ray tube are simultaneously detected by the TES detector.

The overall experimental setup is illustrated in Fig. 4. The following sections describe the experimental configuration, while the details of the data analysis are provided in the Supplementary Materials.

### Muon beam

The experiment used a 25-Hz pulsed muon beam, where each pulse consists of two bunches with a width of ~100 ns and an interval of 600 ns. The beam typically has a Gaussian profile with a transverse size of about 20 mm in σ and is slightly elongated in the vertical direction.

To optimize muon stopping in a solid D_2_ target, we varied the beam momentum and identified a central momentum of 27.5 MeV/*c*, which resulted in a negative muon intensity of ∼10^6^ s^−1^ (*45*).

In this experiment, 5.1 × 10^6^ pulses were irradiated onto a solid D_2_ target, which corresponds to ~57 hours of data acquisition. For background measurements, after the D_2_ target had evaporated, the silver target base was irradiated with 8.2 × 10^5^ pulses (∼9-hour irradiation). As expected, no spectral structure was observed in the background measurement across all energy regions of interest.

### Target

The target configuration is shown in Fig. 4. A solid D_2_ target was prepared by depositing D_2_ gas on a cryogenically cooled silver foil (thickness of 100 μm) from the downstream direction in a vacuum chamber maintained at ~10^−6^ Pa. The silver target base was cooled to 3 K, allowing the gas to solidify upon contact. The resulting solid D_2_ formed a disk-shaped target with a diameter of about 50 mm and a thickness of ~1 mm. To provide thermal shielding, the target was covered with an aluminum shield cooled to 77 K using liquid nitrogen.

### X-ray detector

We used an array of superconducting TES microcalorimeters developed by NIST (*35*). TES-based x-ray spectroscopy has been successfully applied to exotic atom studies at the Paul Scherrer Institute (*46*), the J-PARC Hadron Facility (*47*), and MLF (*48*–*50*). In this experiment, we used TESs with Au absorbers (*51*) instead of those with Bi absorbers (*46*–*50*), which strongly suppress the low-energy tail component in their response functions. The TES system uses a superconducting Mo-Cu bilayer structure, with critical temperature tuned to *T*_*c*_ = 111 to 112 mK. The detector array consists of 192 pixels, each with a Au absorber of size of 340 μm by 340 μm and a thickness of 965 nm. An aperture mask with an opening of size of 280 μm by 280 μm over each absorber was installed to ensure that x-rays hit only the absorbers, which resulted in a total effective detector area of 15.1 mm^2^. In this analysis, ~70 pixels exhibiting low noise and stable performance were selected for each run.

## Supplementary Materials

### This PDF file includes:

Supplementary Text Figs. S1 to S7 References
