# Supplementary material for: Direct observation of muonic molecules in resonance states critical to muon catalyzed fusion
Source: Sci Adv. 2026 Apr 15;12(16):eaed3321. doi: 10.1126/sciadv.aed3321 (PMC13082326; doi:10.1126/sciadv.aed3321)
Supplement: Supplementary file 1 — Supplementary Text Figs. S1 to S7 References [file sciadv.aed3321_sm.pdf]

Science Advances
AAAS

Supplementary Materials for

Direct observation of muonic molecules in resonance states critical to muon catalyzed fusion

Y. Toyama *et al.*

Corresponding author: Y. Toyama, toyama@fsc.chubu.ac.jp; Y. Kino, y.k@tohoku.ac.jp;
S. Okada, sokada@fsc.chubu.ac.jp; T. Yamashita, tyamashita@tohoku.ac.jp

Sci. Adv. **12**, eaed3321 (2026)
DOI: 10.1126/sciadv.aed3321

This PDF file includes:

Supplementary Text
Figs. S1 to S7
References

Data Analysis

X-ray transmission

To block visible and infrared light while maximizing X-ray transmission below 2 keV in the TES detector system, X-ray windows consisting of thin aluminum-coated polymide films (110 nm Al / 200 nm polymide, manufactured by LUXEL Inc.) were used at each cooling stage: 50 K, 3 K, and 50 mK. The TES and target chamber were housed in separate vacuum systems, mechanically decoupled and connected via a helium-gas-filled gap approximately 20 mm thick at 1 atm. A low-energy X-ray filter (LUXEL LEX-HT), made of ultrathin polyimide and aluminum and supported by a mesh, further separated the two vacuums. This configuration minimizes X-ray attenuation while maintaining electrical and vibrational isolation, which is crucial for the TES system's noise sensitivity.

An additional X-ray window with a diameter of 100 mm was installed on the 77 K thermal shield of the target chamber to maintain insulation while allowing the transmission of low-energy X-rays. Windows composed of 2.5 µm thick Mylar with 100 nm aluminum coating on both sides were positioned on the sides of the TES and the X-ray tube. Additionally, 11 µm-thick aluminum foil windows were mounted on the upstream and downstream sides of the 77 K thermal shield, though they did not play a role in the X-ray transmission.

The X-ray transmission coefficient was calculated for photons emitted at the mid-plane of the target, accounting for self-absorption effects. The total transmission curve is shown in Fig. S1, with individual contributions from each layer.

In the transmission spectrum, a K-absorption edge of Al is clearly seen at 1.56 keV in the Al-containing layer. In contrast, Fig. 2 shows a continuous bremsstrahlung background below approximately 1.6 keV, with no apparent discontinuities. This suggests that the background component is not necessarily due to X-rays that have passed through the layers containing Al in the setup.

For the helium gas layer, a purity of 98% was assumed based on experimental conditions, and the remaining 2% was modeled as air. This mixture was used in calculating the transmission curve to reflect the realistic attenuation environment.

The obtained energy dependent transmittance was incorporated into the fit function rather than the spectrum because the obtained spectrum included background events that did not pass through

the X-ray windows.

Pulse processing and selection

TES pulse data were processed by template fitting to extract pulse height information (*52*). Each waveform was recorded as digital data, and the template was constructed by averaging the waveforms of individual pixels. Events affected by background or distorted waveforms, primarily due to charged particle interactions, were excluded from the analysis to ensure accurate energy reconstruction.

Each TES pulse consisted of 1024 sampled points, with the baseline level evaluated using the 256 points preceding the trigger. Figure S2 shows an averaged TES pulse shape used in this analysis. To determine the pulse height, the waveform was fitted around its peak. Although increasing the number of data points generally improves energy resolution, baseline irregularities, such as distortions from thermal fluctuations or extended tails on the falling edge, can degrade performance.

To mitigate these effects, the fitting range was optimized: 16 points out of 256 points were used for baseline evaluation, and 500 points at the falling edge of the pulse were excluded from the fit. As a result a total of $1024 - (240 + 500) = 284$ points were used to determine the pulse height from the full 1024-point waveform.

Timing cut

At J-PARC MLF, the pulsed muon beam arrives in a 25 Hz double-bunch structure, consisting of two 100 ns-wide bunches spaced by 600 ns. Since the TES timing resolution ($\sim$1 µs) is insufficient to resolve individual beam bunches, a single timing gate was applied to separate beam-correlated events from uncorrelated events, such as calibration X-rays.

Figure S3A shows the correlation between TES pulse timing and X-ray energy. The timing offset parameters were tuned after each cooling cycle of adiabatic demagnetization refrigerator (ADR), and the time walk correction was applied after the time offset adjustment. Beam-synchronized X-rays, such as the 2p–1s (2.0 keV) and 3p–1s (2.3 keV) transitions of dµ atoms, are clearly observed. In contrast, calibration X-rays from Al and Cl, which are uncorrelated with the beam, appear as continuous bands at 1.5 keV and 2.6 keV, respectively.

Figure S3B presents the one dimensional projection of Fig. S3A. The extended tail toward later times reflects about the 2 μs lifetime of muons. To improve the signal-to-noise ratio in the ddμ* spectral analysis, events within a time window of −1.0 μs to +1.5 μs relative to the beam trigger were selected.

Calibration and Response function of TES

To ensure precise energy calibration, intense X-rays were required, as each pixel needed independent calibration. Calibration X-rays were continuously monitored during the data acquisition, with energy calibrations performed for each ADR cycle. A tungsten-target X-ray tube generated bremsstrahlung radiation, which irradiated a Al foil and a KCl crystal to produce characteristic X-rays of Al, K, and Cl. These calibration X-rays passed through the thermal shields of the solid D_2 target, reaching the TES detector. The Kα lines of Al (1.5 keV (*53*)), Cl (2.6 keV (*54*)), and K (3.3 keV (*54*)) were used to calibrate the 1.6 keV to 2.0 keV range, and a spline interpolation of three points was used to construct the calibration curve. Figure S4 shows an off-beam spectrum with energy calibration peaks. The referenced X-ray energies for K and Cl correspond to those of elemental K and Cl. The chemical shift due to the KCl compound is estimated to be less than 1 eV (*55*). Therefore, this effect is negligible compared to the influence of the muon beam structure. The response function of TES detectors with Bi absorbers is typically described by the Bortels function (*56*), which models the convolution of a Gaussian with a normalized combination of a delta function and a one-sided exponential tail on the low-energy side (*57*). Although the Au absorber used in this study substantially suppresses the tail component compared to earlier designs, the calibration peaks remain well described by this function, as demonstrated in studies using similar Au absorbers (*51, 58*) and as shown in Fig. S5A.

However, due to the pulsed beam structure at MLF, the detector response differs markedly between in-beam and off-beam conditions. Compared to previous experiments at MLF (*36*), the present setup included a larger amount of material in and around the target, leading not only to energy shifts, but also to noticeable changes in the response function as seen in Fig. S6. Similar effects have been reported in hadron-beam experiments conducted under high counting rates (*59*). These variations are attributed to energetic charged particles, particularly electrons from muon decay, striking the TES array and inducing transient heating of the silicon substrate beneath each

TES pixel. This localized thermal perturbation alters the TES response, as discussed in (*36*).

Therefore, the calibration accuracy was evaluated separately for the in-beam and off-beam spectra. This evaluation was performed against summed spectra of all available pixels after applying pulse and timing cuts.

For the off-beam spectra, the detector response was modeled using the Bortels function. In the case of the Al spectrum, the natural widths and relative intensities of the Kα components were fixed based on known reference values (*53*). These components include Kα_1, Kα_2, Kα_3, Kα_4, Kα_5, Kα_6, Kα', Kα'_3, and Kα_{sat}. Each peak was fitted using a convolution of the instrumental response function to determine the energy resolution. The relative positions of all peaks were constrained based on known reference values (*53*), and only the centroid of the Kα_1 peak was treated as a free parameter.

In total, six free parameters were included in the fit: the amplitude of the peak, the centroid position of Kα_1, the width (i.e., resolution) of the Gaussian peak, the ratio between the peak and tail components, the decay constant (τ) of the tail function, and a constant background level.

For the K and Cl spectra, the peak positions and natural widths of the satellite lines were included as additional fit parameters, since the satellite structures in these compounds are not well characterized.

The energy resolution of the TES detector, as determined from the off-beam spectra, was 5.7 eV (FWHM) at 1.5 keV. Although this is inferior to the intrinsic performance of TES detectors, the degradation is attributed to uncertainties in the satellite X-ray structure of KCl. Though further analysis could improve the result, the current level of accuracy is sufficient for the absolute energy determination required in this study.

It is difficult to directly estimate the in-beam response from the calibration peaks. This is because the muon beam produces signals with a time width of about 1 μs, whereas calibration X-rays are detected continuously at a few thousand counts per second and do not provide statistically meaningful peaks when sliced over such short time windows. In this analysis, the beam timing was defined as $t = 0$, and the in-beam response was first evaluated using calibration X-rays detected within the time window from –250 μs to +250 μs. These in-beam calibration peak shapes were also used to study the energy dependence of the detector response. Figure S5B shows in-beam calibration peaks of Al Kα X-rays. The in-beam peak exhibited a high-energy tail structure not

observed in previous experiments, possibly due to increased background. Several response function models were tested for this fit, and the best results were obtained using sum of a Gaussian function for the peak and an asymmetric Gaussian for the tail.

The response function under the in-beam conditions was ultimately determined from the shape of the dμ 2p-1s X-ray peak generated by the muon beam injection. However, as shown in Figure 2, the dμ 2p-1s peak was contaminated by the X-rays from ddμ*. Therefore there was no straightforward method to evaluate dμ 2p-1s peak shape. To address this, we obtained the in-beam response function through an iterative procedure, starting with the response function obtained from the in-beam calibration peak analysis. The ddμ* spectra were initially convolved with an assumed response function (taken from the in-beam calibration peak evaluation), and the parameters of the response function for the dμ 2p-1s peak were obtained by fitting. This updated response function was subsequently folded into the ddμ* spectrum, and the fitting was repeated. This iterative process was performed three times until the function convolved with ddμ* and the parameters obtained from the fitting converged within the margin of error. Since the tail of the dμ 2p-1s peak extended longer to the high energy side than the calibration peaks, an asymmetric Lorentzian was used for the tail function instead of an asymmetric Gaussian. As a result, the in-beam TES detector resolution was determined to be 8.3 ± 0.1 eV (FWHM). Fig S7 shows the response functions obtained from Al Kα X-ray and from dμ 2p-1s, which was used for fitting the ddμ* spectrum. The in-beam energy calibration accuracy were estimated better than 2 eV from energy shifts of the calibration peaks and the dμ 2p-1s peak.

Systematic uncertainties

Systematic uncertainties in the population of each quantum state of the ddμ* system were summarized in the rightmost column of Table1. The dominant contribution to the overall systematic uncertainty arose from the distribution over total angular momentum quantum number J and the energy calibration. Those systematic uncertainties were evaluated based on the following factors and were defined as the square root of the sum of the squares of each. Regarding the statistical uncertainties, negative correlations of fractions among the neighboring $3d\sigma_g$ states ($v = 0, 1, 2$) contributed to increased statistical uncertainties.

1. J distribution

   As for the distribution of rotational quantum number, J, we assumed that the angular momentum distribution, $W(J)$, follows a statistical weight ratio of $W(J) = 2J + 1$, because the X-ray spectrum changes little with different distributions of J and it is difficult to separate J by fitting the experimental spectrum. This simple form is adopted because the Auger transition occurs mainly by the dipole interaction between the muonic molecule and the electron, which does not appreciably alter the angular momentum distribution generated by the initial resonance molecule formation (Vesman mechanism). The uncertainty from this assumption was examined by changing the distribution of J by hand. We introduced a parameter k in the form

   $$W(J) = (2J + 1)\exp(-kJ(J + 1)) \tag{S1}$$

   to estimate the systematic uncertainty. The parameter k was varied within the range [0,5] and held fixed during the fitting process. The variation in fit results due to changes in k was treated as systematic uncertainty. The typical uncertainty from k was a few percent.

   We considered an angular momentum state with $J \leq 3$. This restriction was motivated not only by computational cost limitations, but also by the fact that changes in the X-ray spectral shape changes less pronounced for $J > 3$. In fact, we also examined the uncertainty arising from this limitation by excluding $J = 3$ components. However, its contribution to the determination of v distribution by fitting was found to be negligible.

2. Energy calibration

   A shift in the absolute energy calibration directly affects the fit of the $\mathrm{dd}\mu^*$ spectrum. To evaluate this, we repeated the fit by shifting the spectrum used in the analysis by ± 2 eV, assuming an uncertainty of 2 eV in the absolute energy calibration. The resulting variation was taken as the systematic error. The typical uncertainty from energy calibration was about one percent.

3. Material transmission

   Systematic uncertainties related to X-ray transmittance through the materials used at each stage were evaluated. The dominant contributions arose from the contamination of air in the

helium gas layer. The uncertainty was estimated by changing the contamination of air from 2% to 25%. The typical uncertainty from the transmission calibration was about one percent.

4. Resonance to bound states transition of ddμ*

   We assumed ddμ* in $2p\pi_u$, $v = 0$, $J = 1$ has a branch of resonance to bound transition with probabilities of 30%. The fit result when this branching ratio was set to 0 was treated as a systematic uncertainty. The typical uncertainty from the resonance to bound state was smaller than one percent.

5. Contribution of $4f\sigma_u$ $v = 0$

   As shown in the Discussion, the $4f\sigma_u$ $v = 0$ state is consistent with the energy level constraints and could be observed in the X-ray spectrum. However, when included in the fitting, it yielded an intensity consistent with zero. The contribution of the $4f\sigma_u$ states to the systematic error in the final fit result was negligible compared to other factors.

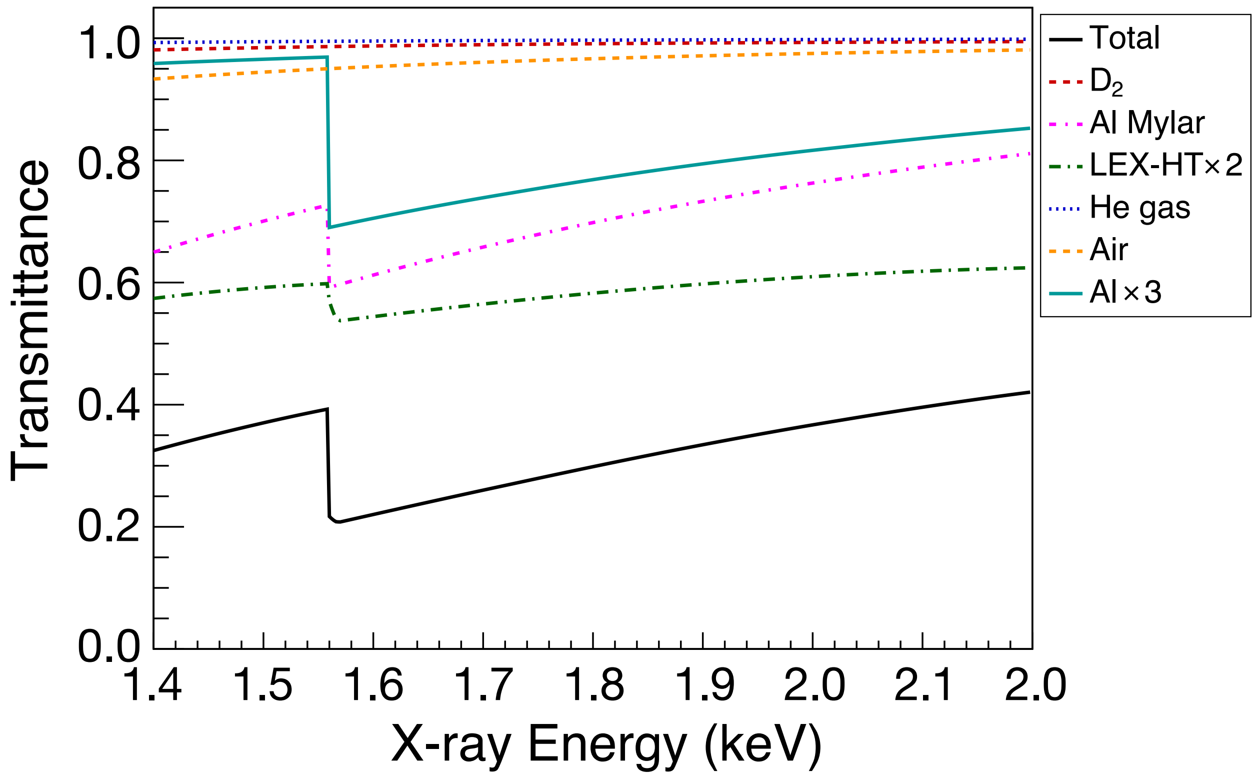

Figure S1: **Simulated X-ray transmission spectrum through the experimental setup.** The total transmission curve (black line) represents the cumulative attenuation caused by all materials positioned between the X-ray source (solid D_2 target) and the TES detector. The individual contributions of each component, including the solid D_2 target, a 77 K thermal shield (aluminized Mylar), dual vacuum windows (LUXEL LEX-HT), a helium gas-filled gap, contaminated air, and three TES radiation shields (thin aluminum foils), are shown separately to highlight their respective impacts on the overall transmission.

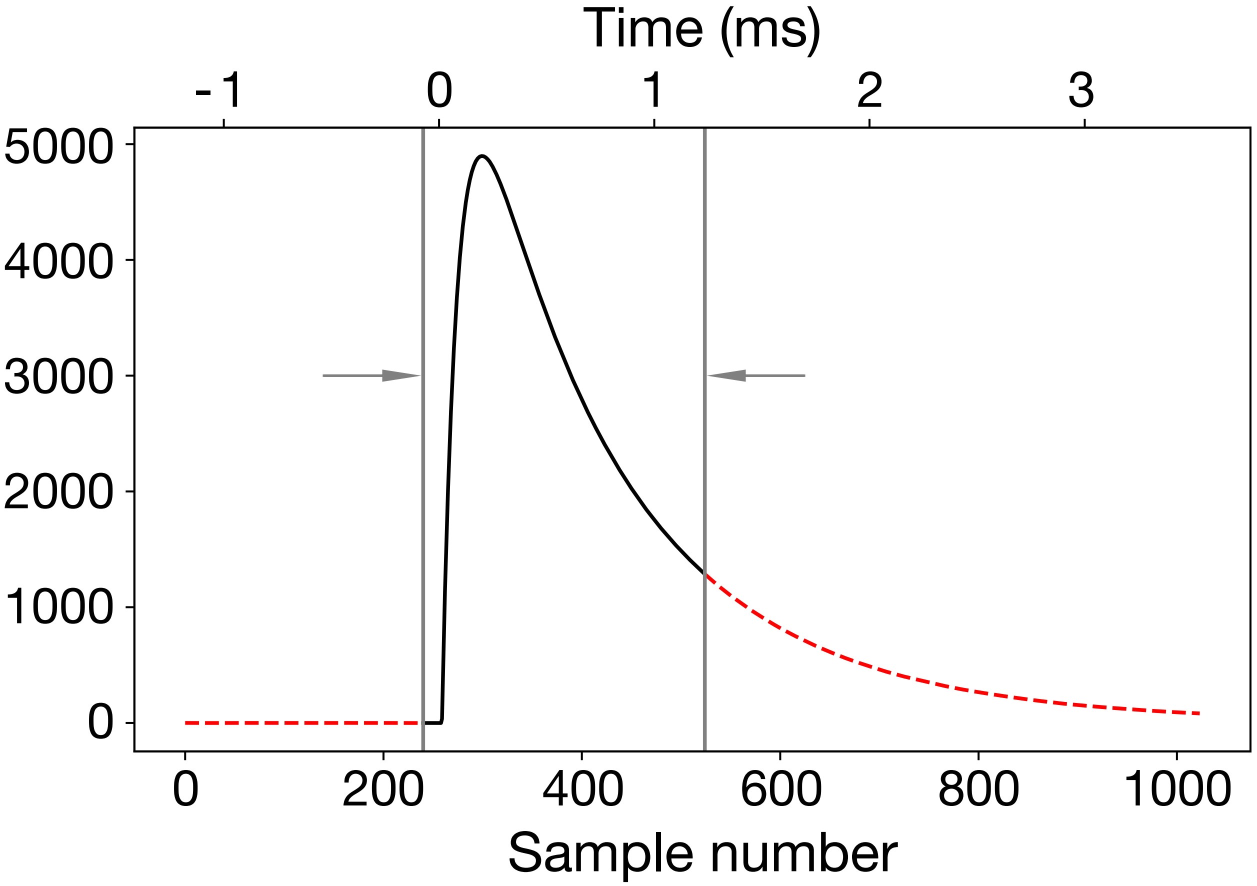

Figure S2: **Averaged pulse shape over 1024 samples for a single TES pixel.** To minimize distortions caused by charged particle hits, only the region indicated by the black solid line was used to extract the pulse height and determine the X-ray energy, excluding the red dashed region from the fit.

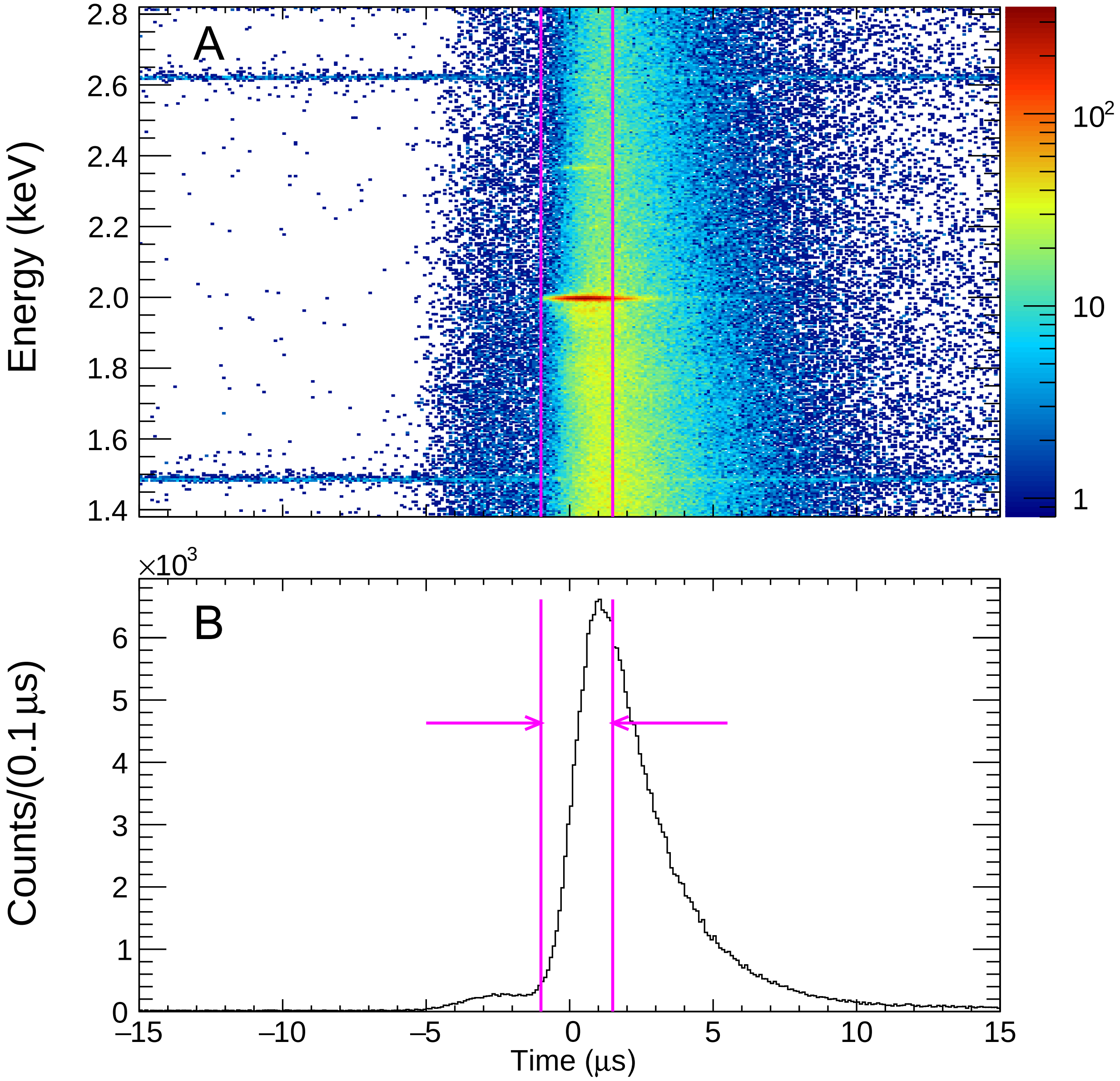

Figure S3: **Time distribution of X-rays relative to the muon beam arrival. A**, Correlation between X-ray energy and arrival time. The 2p-1s X-ray locus of dμ atoms appear near 2 keV. In contrast, Kα X-ray from Al and Cl calibration sources, appear continuously at 1.5 keV and 2.6 keV. **B**, One-dimensional projection of the timing distribution of A. The magenta vertical lines indicate the selected window $t = (-1.0,\ 1.5)$ μs, used to extract beam-correlated ddμ* X-ray events.

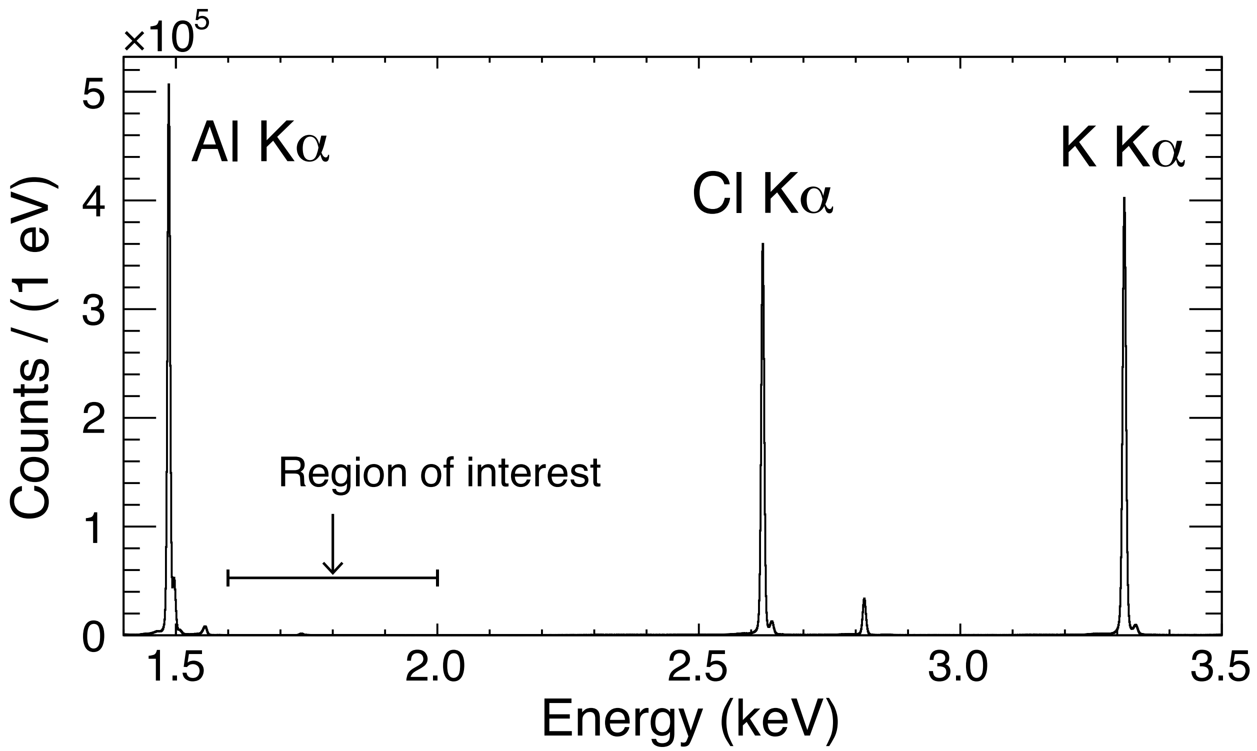

Figure S4: **Energy calibration spectrum acquired without beam, using** Kα **X-rays from Al, Cl, and K generated by an X-ray tube.** These lines served as calibration references. Each TES pixel was independently calibrated, and the spectrum sums over all available calibrated pixels. The marked region of interest indicates the analysis window for beam-synchronized X-rays.

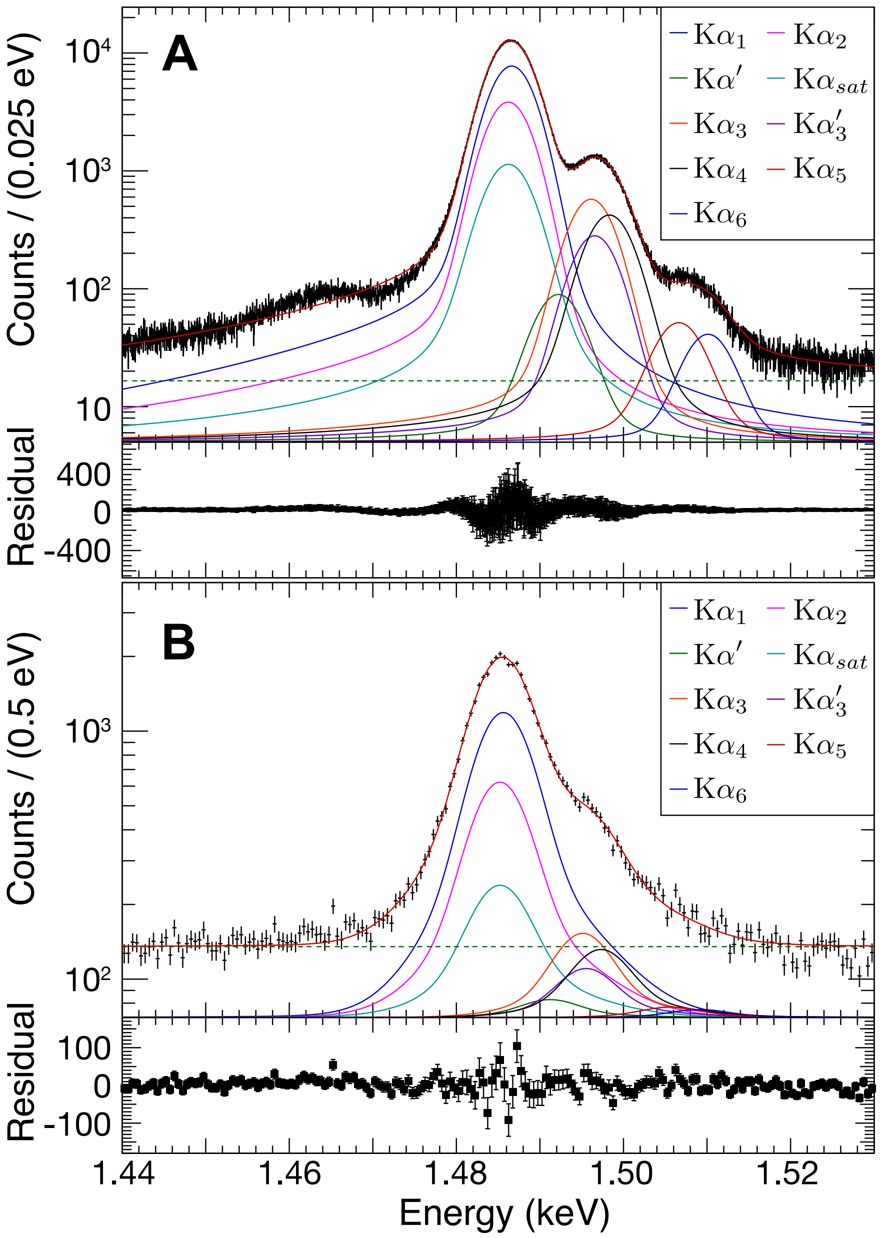

Figure S5: **Calibration peaks of Al** Kα **X-rays.** The red line shows the total fit result, while the colored solid lines represent the fitted components of individual peaks. The dashed line indicates the constant background. **A**, Off-beam spectrum fitted using the sum of a convolution of a Gaussian and a Lorentzian (Voigt) peak function and a tail component modeled by an exponential convolved with a Voigt function. **B**, In-beam spectrum with the time window [-250, 250] μs, fitted using Voigt peaks and an asymmetric Gaussian tail to account for the observed high-energy tail. For clarity, the fitted curves are vertically offset by +5 (off-beam) and +70 (in-beam).

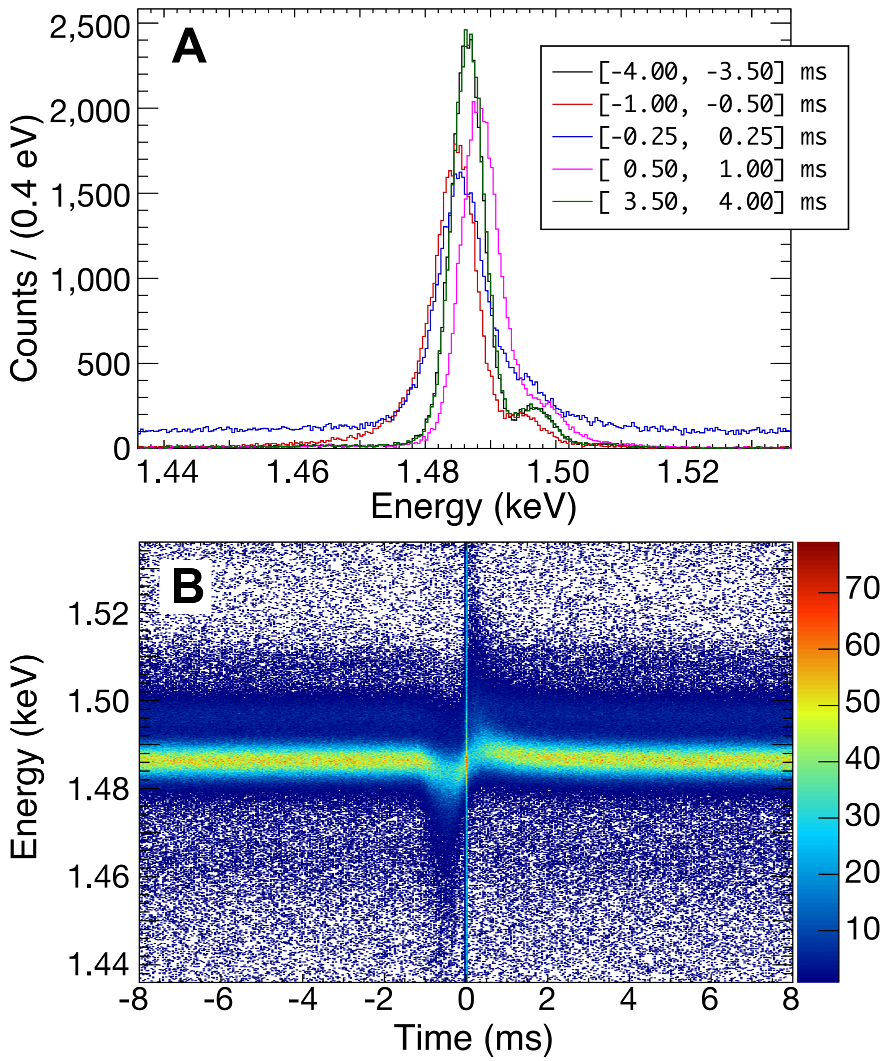

Figure S6: **Time dependence of the Al** Kα **X-ray energy spectra.** $t = 0$ represents muon beam arrival timing. **A**, The one-dimensional histogram of Al Kα X-ray. Peak shapes varied depending on the timing cut conditions. Black and green lines show [-4.00, -3.50] ms and [3.50, 4.00] ms time gate, respectively. They have almost no effect from muon beam irradiation and almost overlaps. **B**, Correlation between energy and timing in the Al Kα X-ray energy region. Energy distortion can be seen in ~ -1 ms to 1 ms region. That corresponds pulse length of the TES.

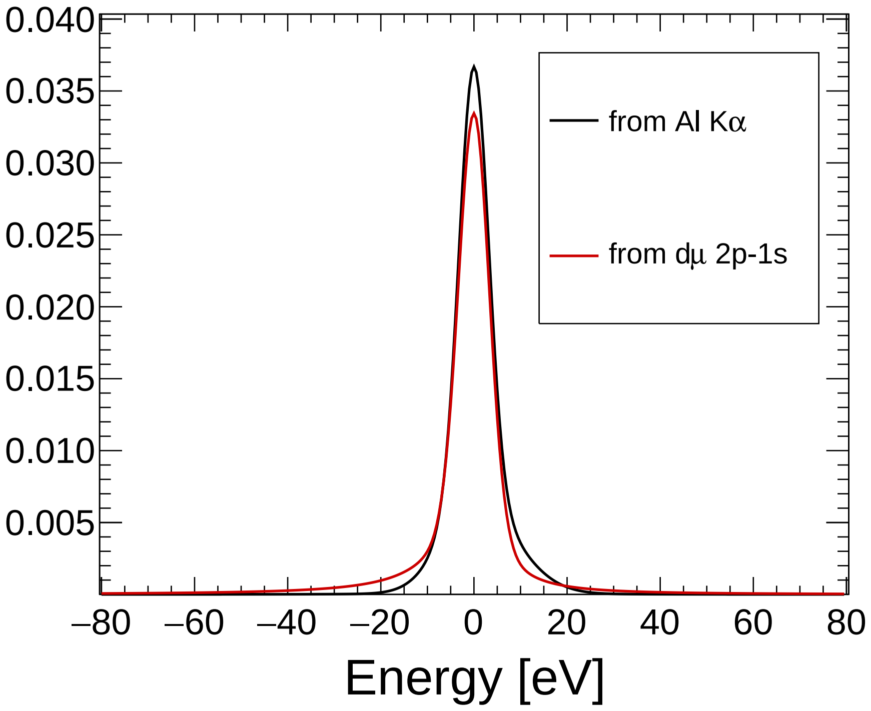

Figure S7: Response functions obtained from Al Kα X-ray and from dμ 2p–1s were shown as black and red solid lines, respectively. Each function was normalized such that its integral equals 1.

REFERENCES

1. F. C. Frank, Hypothetical alternative energy sources for the ‘second meson’ events. *Nature* **160**, 525–527 (1947).

2. A. D. Sakharov, Passive Mesons. *Muon Catalyzed Fusion* (Engl. translation) **4**, 235–239 (1989) [translated from report, Academy of Sciences USSR, P. N. Lebedev Physics Institute (1948)].

3. W. H. Breunlich, P. Kammel, J. S. Cohen, M. Leon, Muon-catalyzed fusion. *Annu. Rev. Nucl. Part. Sci.* **39**, 311–356 (1989).

4. L. I. Ponomarev, Muon catalysed fusion. *Contemp. Phys.* **31**, 219–245 (1990).

5. H. E. Rafelski, D. Harley, G. R. Shin, J. Rafelski, Cold fusion: Muon-catalysed fusion. *J. Phys. B: At. Mol. Opt. Phys.* **24**, 1469–1516 (1991).

6. P. Froelich, Muon catalysed fusion chemical confinement of nuclei within the muonic molecule dt. *Adv. Phys.* **41**, 405–508 (1992).

7. K. Nagamine, M. Kamimura, Muon catalyzed fusion: Interplay between nuclear and atomic physics. *Adv. Nucl. Phys.* **24**, 150–205 (2002).

8. V. R. Bom, A. M. Demin, D. L. Demin, C. W. E. van Eijk, M. P. Faifman, V. V. Filchenkov, A. N. Golubkov, N. N. Grafov, S. K. Grishechkin, K. I. Gritsaj, V. G. Klevtsov, A. D. Konin, A. V. Kuryakin, S. V. Medved’, R. K. Musyaev, V. V. Perevozchikov, A. I. Rudenko, S. M. Sadetsky, Y. I. Vinogradov, A. A. Yukhimchuk, S. A. Yukhimchuk, V. G. Zinov, S. V. Zlatoustovskii, Experimental investigation of muon-catalyzed dt fusion in wide ranges of d/t mixture conditions. *J. Exp. Theo. Phys.* **100**, 663–687 (2005).

9. D. V. Balin, V. A. Ganzha, S. M. Kozlov, E. M. Maev, G. E. Petrov, M. A. Soroka, G. N. Schapkin, G. G. Semenchuk, V. A. Trofimov, A. A. Vasiliev, A. A. Vorobyov, N. I. Voropaev, C. Petitjean, B. Gartner, B. Lauss, J. Marton, J. Zmeskal, T. Case, K. M. Crowe, P. Kammel, F. J. Hartmann, M. P. Faifman, High precision study of muon catalyzed fusion in d2 and hd gas. *Phys. Part. Nucl.* **42**, 185–214 (2011).

10. N. Yamamoto, M. Sato, H. Takano, A. Iiyoshi, Transmutation of LLFP by irradiation of neutrons on muon catalyzed fusion (MCF) reactor. *Plasma Fusion Res.* **16**, 1405074–1405074 (2021).

11. M. Kamimura, Y. Kino, T. Yamashita, Comprehensive study of muon-catalyzed nuclear reaction processes in the *dt*μ molecule. *Phys. Rev. C* **107**, 034607 (2023).

12. Y. Hamada, R. Kitano, R. Matsudo, H. Takaura, M. Yoshida, μTRISTAN. *Prog. Theor. Exp. Phys.* **2022**, 053B02 (2022).

13. K. Nagamine, Generation of ultra-slow muons for large-scale future applications. *Hyperfine Interact.* **103**, 123–135 (1996).

14. S. E. Jones, Muon-catalysed fusion revisited. *Nature* **321**, 127–133 (1986).

15. N. Kawamura, K. Nagamine, T. Matsuzaki, K. Ishida, S. N. Nakamura, Y. Matsuda, M. Tanase, M. Kato, H. Sugai, K. Kudo, N. Takeda, G. H. Eaton, Discovery of temperature-dependent phenomena of muon-catalyzed fusion in solid deuterium and tritium mixtures. *Phys. Rev. Lett.* **90**, 043401 (2003).

16. A. Adamczak, M. P. Faifman, Resonant *dt*μ formation in condensed hydrogen isotopes. *Phys. Rev. A* **72**, 052501 (2005).

17. J. S. Cohen, Capture of negative exotic particles by atoms, ions and molecules. *Rep. Prog. Phys.* **67**, 1769 (2004).

18. E. Vesman, Concerning one possible mechanism of production of the mesic-molecular ion (ddμ). *ZhETF Pisma Redaktsiiu* **5**, 113 (1967).

19. P. Froelich, J. Wallenius, Resonance sidepath in muon catalyzed fusion. *Phys. Rev. Lett.* **75**, 2108–2111 (1995).

20. Y. Kino, M. Kamimura, Muonic atom-nucleus collisions in the energy region of dtμ, resonant states. *Hyperfine Intract.* **101**, 191–196 (1996).

21. T. Yamashita, Y. Kino, K. Okutsu, S. Okada, M. Sato, Roles of resonant muonic molecule in new kinetics model and muon catalyzed fusion in compressed gas. *Sci. Rep.* **12**, 6393 (2022).

22. R. Pohl, H. Daniel, F. J. Hartmann, P. Hauser, F. Kottmann, V. E. Markushin, M. Mühlbauer, C. Petitjean, W. Schott, D. Taqqu, P. Wojciechowski-Grosshauser, Observation of long-lived muonic hydrogen in the *2s* state. *Phys. Rev. Lett.* **97**, 193402 (2006).

23. V. P. Popov, V. N. Pomerantsev, Formation and collisional quenching of the long-lived *2s* state of muonic hydrogen. *Phys. Rev. A* **83**, 032516 (2011).

24. M. Diepold, F. D. Amaro, A. Antognini, F. Biraben, J. M. R. Cardoso, D. S. Covita, A. Dax, S. Dhawan, L. M. P. Fernandes, CREMA Collaboration, Lifetime and population of the *2s* state in muonic hydrogen and deuterium. *Phys. Rev. A* **88**, 042520 (2013).

25. E. Lindroth, J. Wallenius, S. Jonsell, Decay rates of excited muonic molecular ions. *Phys. Rev. A* **68**, 032502 (2003).

26. S. Kilic, J.-P. Karr, L. Hilico, Coulombic and radiative decay rates of the resonances of the exotic molecular ions $pp\mu$, $pp\pi$, $dt\mu$, $dt\pi$, and $dt\mu$. *Phys. Rev. A* **70**, 042506 (2004).

27. T. Yamashita, K. Yasuda, Y. Kino, Radiative decay of muonic molecules in resonance states. *Phys. Rev. A* **111**, 012811 (2025).

28. S. Sakamoto, K. Ishida, K. Nagamine, X-ray studies on muon transfer reaction from excited states of muonic hydrogen atoms to deuterium atoms. *Phys. Lett. A* **260**, 253–261 (1999).

29. M. Augsburger, P. Ackerbauer, W. H. Breunlich, M. Cargnelli, D. Chatellard, J.-P. Egger, B. Gartner, F. J. Hartmann, O. Huot, R. Jacot-Guillarmod, P. Kammel, R. King, P. Knowles, A. Kosak, B. Lauss, J. Marton, M. Mühlbauer, F. Mulhauser, C. Petitjean, W. Prymas, L. A. Schaller, L. Schellenberg, H. Schneuwly, S. Tresch, T. von Egidy, J. Zmeskal, Muon transfer from deuterium to helium. *Phys. Rev. A* **68**, 022712 (2003).

30. B. Lauss, P. Ackerbauer, W. H. Breunlich, B. Gartner, M. Jeitler, P. Kammel, J. Marton, W. Prymas, J. Zmeskal, D. Chatellard, J.-P. Egger, E. Jeannet, H. Daniel, A. Kosak, F. J.

Hartmann, C. Petitjean, Excited state muon transfer in hydrogen/deuterium mixtures. *Phys. Rev. Lett.* **76**, 4693–4696 (1996).

31. W. Higemoto, R. Kadono, N. Kawamura, A. Koda, K. M. Kojima, S. Makimura, S. Matoba, Y. Miyake, K. Shimomura, P. Strasser, Materials and life science experimental facility at the japan proton accelerator research complex iv: The muon facility. *Quantum Beam Sci.* **1**, 11 (2017).

32. P. Strasser, T. Matsuzaki, K. Nagamine, Experimental setup for x-ray spectroscopy of muonic atoms formed from implanted ions in solid hydrogen. *Nucl. Instrum. Methods Phys. Res. A* **460**, 451–456 (2001).

33. K. Okutsu, T. Yamashita, Y. Kino, R. Nakashima, K. Miyashita, K. Yasuda, S. Okada, M. Sato, T. Oka, N. Kawamura, S. Kanda, K. Shimomura, P. Strasser, S. Takeshita, M. Tampo, S. Doiuchi, Y. Nagatani, H. Natori, S. Nishimura, A. D. Pant, Y. Miyake, K. Ishida, Design for detecting recycling muon after muon-catalyzed fusion reaction in solid hydrogen isotope target. *Fusion Eng. Des.* **170**, 112712 (2021).

34. J. N. Ullom, D. A. Bennett, Review of superconducting transition-edge sensors for x-ray and gamma-ray spectroscopy*. *Supercond. Sci. Technol.* **28**, 084003 (2015).

35. W. B. Doriese, P. Abbamonte, B. K. Alpert, D. A. Bennett, E. V. Denison, Y. Fang, D. A. Fischer, C. P. Fitzgerald, J. W. Fowler, J. D. Gard, J. P. Hays-Wehle, G. C. Hilton, C. Jaye, J. L. McChesney, L. Miaja-Avila, K. M. Morgan, Y. I. Joe, G. C. O'Neil, C. D. Reintsema, F. Rodolakis, D. R. Schmidt, H. Tatsuno, J. Uhlig, L. R. Vale, J. N. Ullom, D. S. Swetz, A practical superconducting-microcalorimeter x-ray spectrometer for beamline and laboratory science. *Rev. Sci. Instrum.* **88**, 053108 (2017).

36. T. Okumura, T. Azuma, D. A. Bennett, P. Caradonna, I.-H. Chiu, W. B. Doriese, M. S. Durkin, J. W. Fowler, J. D. Gard, T. Hashimoto, R. Hayakawa, G. C. Hilton, Y. Ichinohe, P. Indelicato, T. Isobe, S. Kanda, M. Katsuragawa, N. Kawamura, Y. Kino, K. Mine, Y. Miyake, K. M. Morgan, K. Ninomiya, H. Noda, G. C. O'Neil, S. Okada, K. Okutsu, T. Osawa, N. Paul, C. D. Reintsema, D. R. Schmidt, K. Shimomura, P. Strasser, H. Suda, D. S. Swetz, T. Takahashi, S. Takeda, S. Takeshita, H. Tatsuno, Y. Ueno, J. N. Ullom, S. Watanabe, S.

Yamada, Dynamical response of transition-edge sensor microcalorimeters to a pulsed charged-particle beam. *IEEE Trans. Appl. Supercond.* **31**, 2101704 (2021).

37. M. C. Fujiwara, A. Adamczak, J. M. Bailey, G. A. Beer, J. L. Beveridge, M. P. Faifman, T. M. Huber, P. Kammel, S. K. Kim, TRIUMF Muonic Hydrogen Collaboration, Resonant formation of *d*μ*t* molecules in deuterium: An atomic beam measurement of muon catalyzed *dt* fusion. *Phys. Rev. Lett.* **85**, 1642–1645 (2000).

38. A. Scrinzi, K. Szalewicz, Auger transition rates for the muonic molecular ion *td*μ. *Phys. Rev. A* **39**, 2855–2861 (1989).

39. V. P. Popov, V. N. Pomerantsev, Collision-induced radiative quenching and other disintegration modes of the *2s* state of muonic hydrogen and deuterium atoms. *Phys. Rev. A* **105**, 042804 (2022).

40. V. E. Markushin, Atomic cascade in muonic hydrogen and the problem of kinetic-energy distribution in the ground state. *Phys. Rev. A* **50**, 1137–1143 (1994).

41. M. Filipowicz, W. Czapliński, E. GuŁa, A. Kravtsov, A. Mikhailov, N. Popov, Energy distributions of excited muonic atoms in deuterium-tritium gas mixtures. *Nuovo Cim. D* **20**, 155–174 (1998).

42. V. P. Popov, V. N. Pomerantsev, Isotopic effects in scattering and kinetics of the atomic cascade of excited $\mu^{-}p$ and $\mu^{-}d$ atoms. *Phys. Rev. A* **95**, 022506 (2017).

43. J. S. Cohen, Stripping and x-ray production in muon-catalyzed d-d and d-t fusion. *Muon Catal. Fusion* **3**, 421–438 (1988).

44. K. Ishida, K. Nagamine, T. Matsuzaki, S. N. Nakamura, N. Kawamura, S. Sakamoto, M. Iwasaki, M. Tanase, M. Kato, K. Kurosawa, H. Sugai, I. Watanabe, K. Kudo, N. Takeda, G. H. Eaton, Measurement of x-rays from muon to alpha sticking and fusion neutrons in solid/liquid d-t mixtures of high tritium concentration. *Hyperfine Intract.* **118**, 203–208 (1999).

45. M. Tampo, K. Hamada, S. Doiuchi, K. Shimomura, Y. Miyake, Beam commissioning of d-line after replacing superconducting solenoid. *KEK Progress Report* **2017-4**, 9 (2017).

46. S. Okada, D. A. Bennett, C. Curceanu, W. B. Doriese, J. W. Fowler, J. D. Gard, F. P. Gustafsson, T. Hashimoto, R. S. Hayano, S. Hirenzaki, J. P. Hays-Wehle, G. C. Hilton, N. Ikeno, M. Iliescu, S. Ishimoto, K. Itahashi, M. Iwasaki, T. Koike, K. Kuwabara, Y. Ma, J. Marton, H. Noda, G. C. O'Neil, H. Outa, C. D. Reintsema, M. Sato, D. R. Schmidt, H. Shi, K. Suzuki, T. Suzuki, D. S. Swetz, H. Tatsuno, J. Uhlig, J. N. Ullom, E. Widmann, S. Yamada, J. Yamagata-Sekihara, J. Zmeskal, First application of superconducting transition-edge sensor microcalorimeters to hadronic atom x-ray spectroscopy. *Prog. Theor. Exp. Phys.* **2016**, 091D01 (2016).

47. T. Hashimoto, S. Aikawa, T. Akaishi, H. Asano, M. Bazzi, D. A. Bennett, M. Berger, D. Bosnar, A. D. Butt, C. Curceanu, W. B. Doriese, M. S. Durkin, Y. Ezoe, J. W. Fowler, H. Fujioka, J. D. Gard, C. Guaraldo, F. P. Gustafsson, C. Han, R. Hayakawa, R. S. Hayano, T. Hayashi, J. P. Hays-Wehle, G. C. Hilton, T. Hiraiwa, M. Hiromoto, Y. Ichinohe, M. Iio, Y. Iizawa, M. Iliescu, S. Ishimoto, Y. Ishisaki, K. Itahashi, M. Iwasaki, Y. Ma, T. Murakami, R. Nagatomi, T. Nishi, H. Noda, H. Noumi, K. Nunomura, G. C. O'Neil, T. Ohashi, H. Ohnishi, S. Okada, H. Outa, K. Piscicchia, C. D. Reintsema, Y. Sada, F. Sakuma, M. Sato, D. R. Schmidt, A. Scordo, M. Sekimoto, H. Shi, K. Shirotori, D. Sirghi, F. Sirghi, K. Suzuki, D. S. Swetz, A. Takamine, K. Tanida, H. Tatsuno, C. Trippl, J. Uhlig, J. N. Ullom, S. Yamada, T. Yamaga, T. Yamazaki, J. Zmeskal, Measurements of strong-interaction effects in kaonic-helium isotopes at sub-ev precision with x-ray microcalorimeters. *Phys. Rev. Lett.* **128**, 112503 (2022).

48. T. Okumura, T. Azuma, D. A. Bennett, P. Caradonna, I. Chiu, W. B. Doriese, M. S. Durkin, J. W. Fowler, J. D. Gard, T. Hashimoto, R. Hayakawa, G. C. Hilton, Y. Ichinohe, P. Indelicato, T. Isobe, S. Kanda, D. Kato, M. Katsuragawa, N. Kawamura, Y. Kino, M. K. Kubo, K. Mine, Y. Miyake, K. M. Morgan, K. Ninomiya, H. Noda, G. C. O'Neil, S. Okada, K. Okutsu, T. Osawa, N. Paul, C. D. Reintsema, D. R. Schmidt, K. Shimomura, P. Strasser, H. Suda, D. S. Swetz, T. Takahashi, S. Takeda, S. Takeshita, M. Tampo, H. Tatsuno, X. M. Tong, Y. Ueno, J. N. Ullom, S. Watanabe, S. Yamada, Deexcitation dynamics of muonic atoms revealed by high-precision spectroscopy of electronic *k* x rays. *Phys. Rev. Lett.* **127**, 053001 (2021).

49. T. Okumura, T. Azuma, D. A. Bennett, I. Chiu, W. B. Doriese, M. S. Durkin, J. W. Fowler, J. D. Gard, T. Hashimoto, R. Hayakawa, G. C. Hilton, Y. Ichinohe, P. Indelicato, T. Isobe, S.

Kanda, M. Katsuragawa, N. Kawamura, Y. Kino, K. Mine, Y. Miyake, K. M. Morgan, K. Ninomiya, H. Noda, G. C. O'Neil, S. Okada, K. Okutsu, N. Paul, C. D. Reintsema, D. R. Schmidt, K. Shimomura, P. Strasser, H. Suda, D. S. Swetz, T. Takahashi, S. Takeda, S. Takeshita, M. Tampo, H. Tatsuno, Y. Ueno, J. N. Ullom, S. Watanabe, S. Yamada, Proof-of-principle experiment for testing strong-field quantum electrodynamics with exotic atoms: High precision x-ray spectroscopy of muonic neon. *Phys. Rev. Lett.* **130**, 173001 (2023).

50. T. Okumura, T. Azuma, D. A. Bennett, W. B. Doriese, M. S. Durkin, J. W. Fowler, J. D. Gard, T. Hashimoto, R. Hayakawa, Y. Ichinohe, P. Indelicato, T. Isobe, S. Kanda, D. Kato, M. Katsuragawa, N. Kawamura, Y. Kino, N. Kominato, Y. Miyake, K. M. Morgan, H. Noda, G. C. O'Neil, S. Okada, K. Okutsu, N. Paul, C. D. Reintsema, T. Sato, D. R. Schmidt, K. Shimomura, P. Strasser, D. S. Swetz, T. Takahashi, S. Takeda, S. Takeshita, M. Tampo, H. Tatsuno, K. Tőkési, X. M. Tong, Y. Toyama, J. N. Ullom, S. Watanabe, S. Yamada, T. Yamashita, Few-electron highly charged muonic ar atoms verified by electronic k x rays. *Phys. Rev. Lett.* **134**, 243001 (2025).

51. P. Szypryt, G. C. O'Neil, E. Takacs, J. N. Tan, S. W. Buechele, A. S. Naing, D. A. Bennett, W. B. Doriese, M. Durkin, J. W. Fowler, J. D. Gard, G. C. Hilton, K. M. Morgan, C. D. Reintsema, D. R. Schmidt, D. S. Swetz, J. N. Ullom, Y. Ralchenko, A transition-edge sensor-based x-ray spectrometer for the study of highly charged ions at the national institute of standards and technology electron beam ion trap. *Rev. Sci. Instrum.* **90**, 123107 (2019).

52. J. W. Fowler, B. K. Alpert, W. B. Doriese, Y.-I. Joe, G. C. O'Neil, J. N. Ullom, D. S. Swetz, The practice of pulse processing. *J. Low Temp. Phys.* **184**, 374–381 (2016).

53. Y. Ménesguen, M.-C. Lépy, Y. Ito, M. Yamashita, S. Fukushima, T. Tochio, M. Polasik, K. Słabkowska, Ł. Syrocki, P. Indelicato, J. P. Gomilsek, J. P. Marques, J. M. Sampaio, J. Machado, P. Amaro, M. Guerra, J. P. Santos, F. Parente, Structure of single KL^0–, double KL^1–, and triple KL^2 ionization in mg, al, and si targets induced by photons, and their absorption spectra. *Radiat. Phys. Chem.* **194**, 110048 (2022).

54. R. D. Deslattes, E. G. Kessler, P. Indelicato, L. de Billy, E. Lindroth, J. Anton, X-ray transition energies: New approach to a comprehensive evaluation. *Rev. Mod. Phys.* **75**, 35–99 (2003).

55. P. E. Best, Electronic structures from x-ray spectra. ii. mostly CLO_3– and CLO_4. *J. Chem. Phys.* **49**, 2797–2805 (1968).

56. G. Bortels, P. Collaers, Analytical function for fitting peaks in alpha-particle spectra from si detectors. *Int. J. Rad. Appl. Instr. A.* **38**, 831–837 (1987).

57. G. C. O'Neil, P. Szypryt, E. Takacs, J. N. Tan, S. W. Buechele, A. S. Naing, Y. I. Joe, D. Swetz, D. R. Schmidt, W. B. Doriese, J. D. Gard, C. D. Reintsema, J. N. Ullom, J. S. Villarrubia, Y. Ralchenko, On low-energy tail distortions in the detector response function of x-ray microcalorimeter spectrometers. *J. Low Temp. Phys.* **199**, 1046–1054 (2020).

58. D. Yan, R. Divan, L. M. Gades, P. Kenesei, T. J. Madden, A. Miceli, J.-S. Park, U. M. Patel, O. Quaranta, H. Sharma, D. A. Bennett, W. B. Doriese, J. W. Fowler, J. D. Gard, J. P. Hays-Wehle, K. M. Morgan, D. R. Schmidt, D. S. Swetz, J. N. Ullom, Eliminating the non-Gaussian spectral response of X-ray absorbers for transition-edge sensors. *Appl. Phys. Lett.* **111**, 192602 (2017).

59. T. Hashimoto, M. Bazzi, D. A. Bennett, C. Berucci, D. Bosnar, C. Curceanu, W. B. Doriese, J. W. Fowler, H. Fujioka, C. Guaraldo, F. Parnefjord Gustafsson, R. Hayakawa, R. S. Hayano, J. P. Hays-Wehle, G. C. Hilton, T. Hiraiwa, Y. Ichinohe, M. Iio, M. Iliescu, S. Ishimoto, Y. Ishisaki, K. Itahashi, M. Iwasaki, Y. Ma, H. Noda, H. Noumi, G. C. O'Neil, H. Ohnishi, S. Okada, H. Outa, K. Piscicchia, C. D. Reintsema, Y. Sada, F. Sakuma, M. Sato, D. R. Schmidt, A. Scordo, M. Sekimoto, H. Shi, D. Sirghi, F. Sirghi, K. Suzuki, D. S. Swetz, K. Tanida, H. Tatsuno, M. Tokuda, J. Uhlig, J. N. Ullom, S. Yamada, T. Yamazaki, J. Zmeskal, Beamline test of a transition-edge-sensor spectrometer in preparation for kaonic-atom measurements. *IEEE Trans. Appl. Supercond.* **27**, 2100905 (2017).
